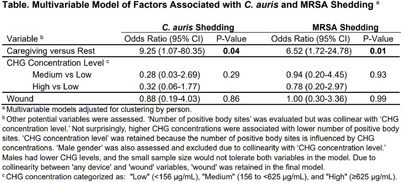# Candida auris and MRSA Shedding During Caregiving versus Rest in Nursing Homes

**DOI:** 10.1017/ash.2025.351

**Published:** 2025-09-24

**Authors:** Gabrielle Gussin, Raveena D. Singh, Julie Shimabukuro, Raheeb Saavedra, Akhil Patel, Steven Vu, Cassiana Bittencourt, Susan Huang

**Affiliations:** 1 University of California, Irvine; 2 University of California, Irvine School of Medicine; 3 University of California Irvine Health; 4 Employee; 5 UCI

## Abstract

**Background:** Candida auris and methicillin-resistant Staphylococcus aureus (MRSA) are prevalent in nursing homes, and both are known to shed profusely from the skin. We evaluated the degree of differential shedding during caregiving activities versus at rest in nursing home residents. **Methods:** Residents at two nursing homes were screened for C. auris and MRSA using nares, axilla/groin, and peri-rectal swabs. Carriers of C. auris, some of whom also carried MRSA, were evaluated for proximal shed around their bed during rest and caregiving activities using chromogenic settle plates. Morning caregiving activities (e.g. hygiene care, linen/clothing change) were noted to generally take 12 minutes. For rest, settle plates were placed for a 12-minute period prior to the resident awakening in the morning. For caregiving, settle plates were placed for the 12-minute period of morning activity shortly after awakening. Twin rest-caregiving measurements were taken on three separate days per C. auris carrier. In addition, prior to caregiving, bilateral nares, hands, axilla, groin, and perirectal swabs were taken for C. auris and MRSA culture, along with an axilla/groin swab for measuring chlorhexidine concentration (CHG used for routine bathing). Logistic regression with person-level clustering analyzed associations between positive settle plates (“shedding”) and activity (caregiving versus rest), along with other adjusters. **Results:** The study included 23 C. auris carriers, 15 of whom carried MRSA. 65% were male, 91% had an indwelling device, 39% had wounds. Mean number of positive body sites was 2.3 for C. auris and 1.2 for MRSA. Median CHG concentration was 156 µg/mL (IQR=39-1250). Shedding occurred more frequently during caregiving versus rest for both C. auris (8/69 vs 1/69, P=0.02) and MRSA (15/69 vs 3/69, p=0.002). In multivariable models (Table), caregiving was associated with increased odds of shedding for both C. auris (OR: 9.25 (95% CI: 1.07-80.35), P=0.04) and MRSA (OR: 6.52 (95% CI: 1.72-24.78), P =0.01). Higher CHG concentrations were non-significantly associated with reduced shedding of both pathogens. **Conclusion:** C. auris and MRSA shedding increased significantly during caregiving activities, supporting CDC’s current recommendations for enhanced barrier precautions in nursing homes, which involve gown and glove use during high-contact care for carriers of multidrug-resistant organisms. Remarkably, shedding was readily detected within 12 minutes of morning caregiving, highlighting a rapid “plume effect” during resident care.

**Figure:**